# Adequate sensing of ventricular fibrillation?

**DOI:** 10.1007/s12471-017-0995-y

**Published:** 2017-04-26

**Authors:** A. W. G. J. Oomen, B. M. van Gelder, F. A. L. E. Bracke

**Affiliations:** Catharina Hospital, Eindhoven, The Netherlands

We present a case of an 81-year-old female patient who was resuscitated after out-of-hospital ventricular fibrillation. Clinical recovery was fine, but cardiological analysis showed a dilated cardiomyopathy with a poor systolic left ventricular function, left bundle branch block and persistent atrial fibrillation. There was no significant coronary artery disease. We prescribed heart failure medication and implanted a biventricular implantable cardioverter-defibrillator (ICD – Medtronic Viva Quad XT CRT-D).

Despite medication, atrial fibrillation with a high ventricular rate persisted. To ensure adequate biventricular pacing, she underwent a successful atrioventricular node ablation. Because of a relative low amplitude R wave in the RV tip to RV ring configuration, RV sensing was programmed to the RV tip to RV coil configuration. A few weeks later, she presented at the emergency department with recurrent complaints of dizziness and lightheadedness. ICD interrogation showed several episodes as shown in Fig. 1 and 2.

**Fig. 1:**
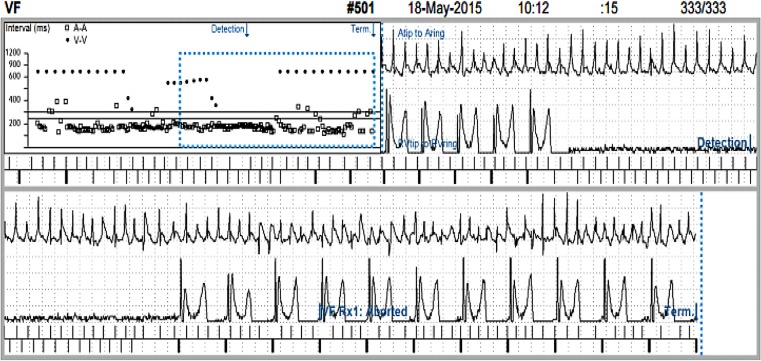
Detection of a fast ventricular tachycardia/ventricular fibrillation that terminated spontaneously. Upper tracing is the atrial electrogram, middle tracing the ventricular electrogram, lower tracing the marker channel

**Fig. 2:**
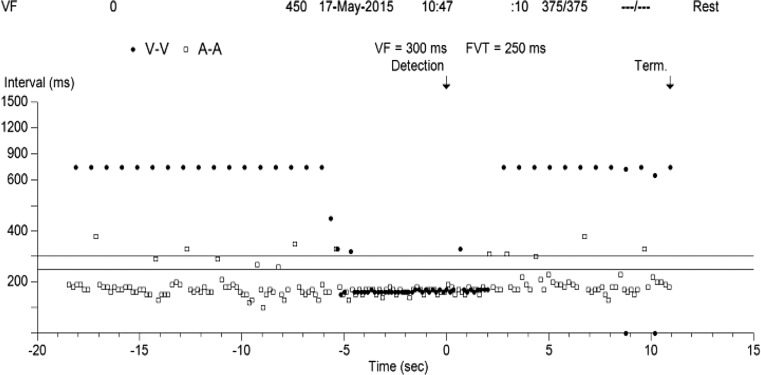
Interval plot with V‑V and A‑A intervals of a similar episode as demonstrated in Fig. 1

What could be the explanation of this phenomenon?

## Answer

You will find the answer elsewhere in this issue.

